# 3D-Printed Hydrogels in Orthopedics: Developments, Limitations, and Perspectives

**DOI:** 10.3389/fbioe.2022.845342

**Published:** 2022-04-01

**Authors:** Zhen Liu, Weiwei Xin, Jindou Ji, Jialian Xu, Liangjun Zheng, Xinhua Qu, Bing Yue

**Affiliations:** ^1^ Department of Bone and Joint Surgery, Department of Orthopedics, Renji Hospital, School of Medicine, Shanghai Jiao Tong University, Shanghai, China; ^2^ The First Clinical Medical College, Shandong University of Traditional Chinese Medicine, Jinan, China

**Keywords:** bone joint repair, reconstructive implant, antibacterial and anti-infection characteristics, 3D printing, hydrogel

## Abstract

Three-dimensional (3D) printing has been used in medical research and practice for several years. Various aspects can affect the finished product of 3D printing, and it has been observed that the impact of the raw materials used for 3D printing is unique. Currently, hydrogels, including various natural and synthetic materials, are the most biologically and physically advantageous biological raw materials, and their use in orthopedics has increased considerably in recent years. 3D-printed hydrogels can be used in the construction of extracellular matrix during 3D printing processes. In addition to providing sufficient space structure for osteogenesis and chondrogenesis, hydrogels have shown positive effects on osteogenic and chondrogenic signaling pathways, promoting tissue repair in various dimensions. 3D-printed hydrogels are currently attracting extensive attention for the treatment of bone and joint injuries owing to the above-mentioned significant advantages. Furthermore, hydrogels have been recently used in infection prevention because of their antiseptic impact during the perioperative period. However, there are a few shortcomings associated with hydrogels including difficulty in getting rid of the constraints of the frame, poor mechanical strength, and burst release of loadings. These drawbacks could be overcome by combining 3D printing technology and novel hydrogel material through a multi-disciplinary approach. In this review, we provide a brief description and summary of the unique advantages of 3D printing technology in the field of orthopedics. In addition, some 3D printable hydrogels possessing prominent features, along with the key scope for their applications in bone joint repair, reconstruction, and antibacterial performance, are discussed to highlight the considerable prospects of hydrogels in the field of orthopedics.

## 1 Introduction

Without effective intervention and treatment, existing injuries and defects can lead to new complications, such as severe infections, which can subsequently result in severe necrosis of the bone and joint, and ultimately the complete loss of bone and joint function. Existing clinical approaches to bone and joint surgery include allogeneic and autologous bone grafts and metal substitute grafts (Levato et al., 2017), with autologous bone grafts being limited by the scarcity of donors; allogeneic bone grafts are limited in that they may lead to immune rejection. A significant issue associated with metal substitute grafts is that they are prone to wear and tear. Therefore, the life span of metal substitutes is generally short and there is a possibility that the individual may have to undergo secondary surgery. In fact, according to the literature (Canovas and Dagneaux, 2018), a survey showed that up to 30% of patients were dissatisfied after total knee arthroplasty.

Hydrogel-based three-dimensional (3D) printing technology has emerged as an effective treatment option for bone and joint injury problems, and its comprehensive advantages are being gradually unfolded. 3D-printed hydrogel materials not only possess most repair properties of existing repair materials (Perera et al., 2021), but also have unique ductility, hydrophilicity, and histocompatibility; moreover, there are extensive material choices as a considerable number of 3D-printed hydrogel materials have been discovered. Thus, owing to their unique advantages, hydrogels hold a huge potential in bone and joint tissue repair, anti-infection, and even tumor treatment.

From the 1990s to the 2010s, researchers experimented with various properties of 3D-printed hydrogels in the laboratory; thus, there has been significant progress in hydrogel-based 3D printing technology. In this paper, we will briefly introduce 3D-printed hydrogels and summarize the main and latest scientific findings in the field of 3D hydrogels in orthopedics to help gain a holistic and intuitive understanding of all aspects of 3D-printed hydrogels. The existing 3D printing technologies applicable to hydrogel materials are also presented. This review not only demonstrates the progress and technologies that have been achieved but also reflects on the problems that exist in order to promote the advancement of hydrogel-based 3D printing technology in the field of orthopedics and its early application in the practical treatment of bone and joint tissue repair.

## 2 Hydrogels

### 2.1 Introduction to Hydrogels

Currently, hydrogels are popular biomaterials for 3D printing and are considered to be the core of 3D bioprinting. They are 3D mesh structures that are composed of highly absorbent cross-linked polymer chains, which swell rapidly in water and retain a large amount of water without dissolving in the swollen state (Abdollahiyan et al., 2020). They are cross-linked by physicochemical means, and the degree of cross-linking determines the water absorption; the higher the degree of cross-linking, the lower the water absorption. Therefore, the water content of hydrogels can vary considerably, from less than 10% to more than 95%, which makes it possible for hydrogels to exhibit both solid and liquid behavior (Gungor-Ozkerim et al., 2018). The solid nature of the hydrogel allows it to maintain a certain volume and form under certain conditions, whereas the liquid nature refers to its ability to allow solutes to diffuse and permeate through the hydrogel. The ability to maintain a certain volume and morphology ensures the feasibility of hydrogels as 3D printing scaffold materials (Turnbull et al., 2018), whereas the ability to allow solutes to diffuse within it makes it possible to add various inorganic and organic solutes to hydrogels.

### 2.2 Hydrogel Classification

#### 2.2.1 Macro and Micro Hydrogels

Hydrogels can be classified according to their size and shape into macroscopic and microscopic gels (microspheres) (Nikolova and Chavali, 2019). Macroscopic hydrogels can be further classified into columnar, porous sponge, fibrous, membrane, spherical, etc. according to their shapes, while microscopic hydrogels can be classified into micron and nanoscale gels according to their diameters.

Nanocomposite biomaterials are a relatively new class of materials that combine biopolymers and biodegradable matrix structures with nanoscale bioactive and easily absorbed fillers (Bharadwaz and Jayasuriya, 2020). The nanofillers induced into polymeric matrices present important physical and chemical properties, such as large surface area, improved mechanical strength and stability, improved cell adhesion, proliferation and differentiation, and tissue regeneration compatibility for biomaterials (Zhai et al., 2017).

#### 2.2.2 External Stimulation

Hydrogels can be divided into two categories according to their response to external stimuli: conventional and environment-sensitive hydrogels. Conventional hydrogels are insensitive to changes in the environment, such as temperature or pH. Environment-sensitive hydrogels are a class of polymer gels that can sense small changes or stimuli in the external environment (such as temperature, pH, light, electricity, pressure, etc.) and produce corresponding changes in physical structure and chemical properties or even mutations (Zhang et al., 2021). An impressive feature of such hydrogels is that their solvation behavior changes significantly during the response to the environment; this stimulus response property enables these hydrogels to be used as sensors, controlled release switches, etc., which is the focus of considerable current research (Jing et al., 2021).

An example is a hydrogel with a photothermal agent as its component, which converts light energy into heat under near infrared (NIR) irradiation. Its target temperature can be adjusted by changing the concentration and ratio of the photothermal agent, the irradiation time, and the laser intensity (Zhang et al., 2021). Mild local heat (41–43 °C) promotes cell proliferation, angiogenesis, wound healing, and bone regeneration, while higher temperatures (>50 °C) can effectively inhibit bacterial proliferation in infected wounds (Ji et al., 2020). Therefore, the photothermal effect can be adapted to different applications by using different temperatures.

#### 2.2.3 Constituent Materials

Based on materials, hydrogels can be divided into natural polymeric hydrogels and synthetic polymeric hydrogels. The primary natural materials are currently agarose, alginate, gelatin, hyaluronic acid, and fibrin, and the main artificial materials are polyvinyl alcohol and polyethylene glycol (Naranda et al., 2021).

Polysaccharide hydrogel materials (Sahai et al., 2021) are carbohydrate macromolecules that are constructed by repeating monosaccharide units linked by glycosidic bonds. The family of polysaccharide hydrogels is extremely large; it includes agarose, chitin (Demirtas et al., 2017; Yadav et al., 2021), heparin, chondroitin sulfate, etc. Gelatin is a product of collagen degradation. It is cost-effective and promotes cartilage and bone regeneration. Hyaluronic acid is an important component of the extracellular matrix of cartilage, and it effectively activates signaling pathways that promote cell differentiation and proliferation, thus enabling efficient cartilage or bone repair and reconstruction (Haung et al., 2021).

Natural polymers have better biocompatibility and environmental sensitivity, and they are abundantly available and inexpensive compared to artificial materials (Wang et al., 2019). However, natural polymers have lesser stability and are prone to degradation. Synthetic polymer hydrogels, conversely, have relatively better stability and mechanical strength. We have summarized the emerging 3D-printed composite hydrogels enlisted in this paper in Table 1.

**TABLE 1:** Properties of typical composite 3D-printed hydrogels.

Hydrogel composition	Cross-linking method	3D printing method	Generating organizations	Advantages	Limitations	References
Hyaluronic acid (HA)/alginate/polylactic acid (PLA)	Physical cross-linking	Extrusion-based printing method/droplet-based printing	Cartilage	Biocompatibility	Low mechanical strength	Bio-inspired hydrogel composed of hyaluronic acid and alginate as a potential bioink for 3D bioprinting of articular cartilage engineering constructs. Acta Biomater. 2020 Apr

Methacrylated poly [N-(2-hydroxypropyl)methacrylamide mono/diacrylate] (pHPMA-lac)/polyethylene glycol (PEG)/methacrylated hyaluronic acid (HAMA)/polycaprolactone (PCL) co-printing	Chemical cross-linking	Extrusion-based printing	Cartilage	Biocompatibility	Higher HAMA concentrations are detrimental to chondrogenesis	Development of a thermosensitive HAMA-containing bio-ink for the fabrication of composite cartilage repair constructs. Biofabrication. 2017 Mar

Alginate/Gelatin/Chondroitin Sulfate/Graphene Oxide Nano	Physical cross-linking, chemical cross-linking	Extrusion-based printing (micro-extrusion)	Cartilage	Printability	High preparation cost	Chondroinductive Alginate-Based Hydrogels Having Graphene Oxide for 3D-Printed Scaffold Fabrication. ACS Appl Mater Interfaces. 2020 Jan

Gelatin/alginate/nanosilicate	Physical cross-linking	Extrusion-based printing	Bone	Mechanical strength	--	3D-bioprinted functional and biomimetic hydrogel scaffolds incorporated with nanosilicates to promote bone healing in rat calvarial defect model. Mater Sci Eng C Mater Biol Appl. 2020 Ju

Gelatin methacrylate (GelMA)/vascular endothelial generating factor (VEGF)/silicate nanosheets	Chemical cross-linking	Extrusion-based printing (stitching of cylindrical structures)	Bone	Promotes bone tissue production capacity	Complex printing process	Bioprinted Osteogenic and Vasculogenic Patterns for Engineering 3D Bone Tissue. adv Healthc Mater. 2017

Silk protein/nano hydroxyapatite/nano silver ion/nano gold ion	Chemical cross-linking	Extrusion-based printing	Bone	Antibacterial activity	Higher cost of raw materials	Antibacterial silk fibroin/nanohydroxyapatite hydrogels with silver and gold nanoparticles for bone regeneration. nanomedicine. 2017 Jan
Hydroxyapatite (HA)/polydopamine (PDA)/carboxymethyl chitosan (CMCS)	Chemical cross-linking	Extrusion-based printing	Bone	Osteogenesis	--	Bifunctional scaffolds of hydroxyapatite/poly (dopamine)/carboxymethyl chitosan with osteogenesis and anti-osteosarcoma effect. Biomater Sci. 2021 May

Alginate/Methylcellulose/Magnesium silicate clay (Laponite)	Physical cross-linking	Extrusion-based printing	Bone/Cartilage	Drug release effect	--	Development of a clay based bioink for 3D cell printing for skeletal application. Biofabrication. 2017 Jul
Chondroitin methacrylate-sulfate (CSMA)/thermosensitive poly (N-(2-hydroxypropyl)methacrylate-mono/diacrylate)/polyethylene glycol triblock copolymer (MP)	Chemical cross-linking	Extrusion-based printing (microvalve)	Cartilage	Thermal Controllability	Difficult to apply clinically	A thermo-responsive and photo-polymerizable chondroitin sulfate-based hydrogel for 3D printing applications. Carbohydr Polym. 2016 Sep

Hydrogel materials composed of decellularized extracellular matrix have recently attracted significant attention in the experimental field. The decellularized extracellular matrix contains almost all the natural materials for the microenvironment required for cell growth (Kabirian and Mozafari, 2020). Furthermore, the matrix is loose and porous with extremely superior biocompatibility and degradability. Qiu et al. investigated decellularized extracellular matrix hydrogel materials based on the periosteal matrix (PEM) (Qiu et al., 2020). This matrix is a highly vascularized tissue that is deeply involved in the bone healing process; previous studies have shown that bio-scaffolds composed of periosteal matrix undergo spontaneous biomineralization and play an active role in bone defect repair. Hydrogels derived from a porcine decellularized periosteum were generated and characterized. The experimental results indicate that this hydrogel material promoted macrophage chemotaxis and M2 polarization associated with constructive bone remodeling and did not trigger adverse immune responses. It also promotes vascular formation and migration, osteogenic differentiation, and bone biomineralization. This illustrates the dynamic involvement of PEM hydrogels in all phases of the fracture repair process, thereby promoting bone regeneration *in vivo*.

#### 2.2.1 Cross-Linking Methods

**FIGURE 1:**
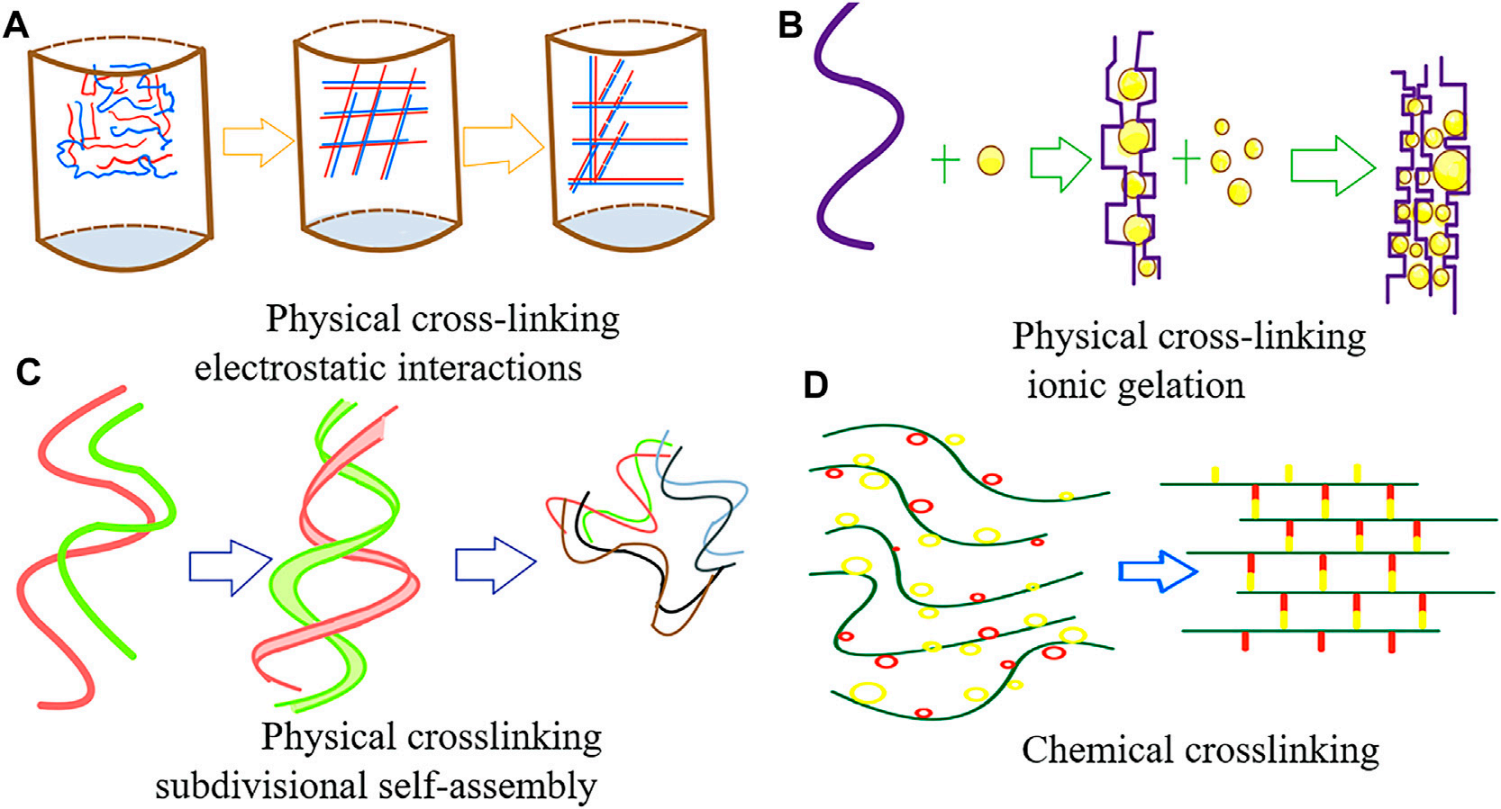
Cross-linking methods for hydrogels.

Hydrogels can be classified into physical and chemical gels based on the cross-linking method of the hydrogel. Physical gels are hydrogels that are formed by cross-linking through physical forces such as electrostatic interactions, hydrogen bonding, hydrophobic interactions, and other non-covalent bonds (Unagolla and Jayasuriya, 2020), which are non-permanent and mostly reversible. Thus, they are also known as pseudogels. However, many natural polymers, such as agar, can permanently maintain this gel state at room temperature. Among synthetic polymers, polyvinyl alcohol (PVA) also possesses such characteristics After the freeze–thaw treatment, PVA hydrogels that remain stable below 60°C can be obtained (Suzuki and Sasaki, 2015) (Figure 1).

Chemical cross-linking occurs mainly through covalent bonding and free-radical polymerization to form the 3D structure of the hydrogels. The cross-linking methods mainly include free radical polymerization, thiol-based Michael reactions, Schiff base reactions, and enzymatic cross-linking reactions. All these cross-linking methods are permanent (Unagolla and Jayasuriya, 2020), and thus, chemically cross-linked hydrogels are also called true gels.

Currently, the cross-linking methods most commonly used in printing go beyond the above-mentioned methods (Zhao et al., 2021). For example, methylated hydrogels with photosensitizers can rapidly form cross-links under blue light and UV light and thus gelatinize. Furthermore, sodium alginate can swiftly be cross-linked and gelatinized in a calcium chloride solution. Environmentally sensitive hydrogels are also capable of self-crosslinking in response to changes in external conditions. For instance, a temperature-sensitive hydrogel that is in a liquid state when the temperature is above 25 °C and rapidly gelatinizes when the temperature falls below 25 °C, has been reported.

Currently, the trendy research direction is composite hydrogel materials, which usually involve multiple types of crosslinking mechanisms. In this way, hydrogels can be fabricated with the desired properties. For example, the crosslinking of GelMA/alginate bioinks involves free radical polymerization of GelMA and electrostatic interactions of alginate (Stanco et al., 2020)to form interpenetrating networks.

### 2.3 Basic Properties of Hydrogels

#### 2.3.1 Biocompatibility and Degradability

Biocompatibility is a significant factor for 3D bioprinting materials. It refers to the functional capabilities of a hydrogel material *in vivo* and *in vitro* without causing any adverse biological reactions (You et al., 2017). Specifically, a biocompatible material does not produce any toxic side effects on the cells and the body, does not cause hypersensitivity in the body, and does not produce toxic by-products when degraded *in vivo* and *in vitro*. Some properties of the 3D-printed hydrogels such as material temperature could be changed throughout the entire 3D printing process (Hölzl et al., 2016); the biocompatibility of the material during the entire process under different conditions is another important criterion to consider. Under the modulation of the experimental means of the experimental staff, it is desirable that the 3D-printed hydrogels are decomposed and reabsorbed *in vivo*. Degradation depends mainly on volumetric dissolution based on several mechanisms, such as hydrolysis (ester or enzyme), photolysis, untwisting, and combinations of these mechanisms. The degradation of 3D-printed hydrogel materials creates more space for cell proliferation (Ouyang et al., 2016), migration, and vascular infiltration.

#### 2.3.2 Mechanical Strength

The mechanical strengths of hydrogels can be considered based on many different perspectives. In some cases, tissue formation mainly depends on the mechanical properties of the hydrogel. Hydrogel materials should ideally possess good mechanical strength throughout the 3D printing process. The rheological properties in printing will be discussed specifically in the next section. The mechanical strength here refers mainly to the ability to maintain its own structure after printing (Gómez et al., 2016). Hydrogel materials are exposed to pressures and stresses from all angles, whether they are *in vitro* or later implanted in the body, and therefore, they need to have good resistance to compression. In addition to the compressive capacity, tensile resistance is essential (Yue et al., 2020).

For example, the human knee joint cartilage–bone interface can bear compressive stresses of 1 MPa and an interfacial toughness of approximately 800 J m^−2^ over 1 million cycles of loads per year (Zhao et al., 2021). In order for the hydrogel structure to attain this strength, a few methods have been used, including supplementing the hydrogel with mineral particles such as hydroxyapatite to produce a composite hydrogel, co-printing by combining printed polycaprolactone (PCL) scaffolds (Mouser et al., 2017), and exploiting cross-linking mechanisms (Bi et al., 2019). For example, alginate hydrogels are cross-linked using a three-step process to improve elastic stiffness.

#### 2.3.3 Viscosity

Viscosity refers to the intrinsic resistance of a fluid to flow in response to an external force. The molecular weight, concentration, and prevailing temperature of a hydrogel together determine its viscosity. In general, the higher the molecular weight and concentration, the higher is the corresponding viscosity (Han et al., 2021). Particularly, in extrusion-based 3D bioprinting, the hydrogel should possess a high viscosity, because it will help counteract the surface tension of the hydrogel. Further, the hydrogel droplets can be extruded more smoothly from the nozzle and further form continuous lines (Daly et al., 2017), which ensures that the two adjacent hydrogel chains do not bond together. However, high concentrations of hydrogels cannot form a good extracellular microenvironment, and thus, low concentrations of high-molecular-weight hydrogels are preferable.

#### 2.3.4 Shear Dilution

Shear dilution refers to the ability to decrease viscosity as the shear rate increases. Normally, the higher the concentration of the hydrogel system, the more pronounced is the shear dilution. Hydrogels are exposed to high shear rates from within the device during 3D bioprinting, and the viscosity decreases, which ensures the survival of cells and therefore, the adaptability of 3D bioprinting.

#### 2.3.5 Printability

Most existing 3D bioprinting techniques adopt a layer-by-layer approach, and thus, it is necessary that hydrogel materials maintain their original structure and morphology during the entire process of 3D printing (Kyle et al., 2017). This means that they need to have structural fidelity and integrity. This property may be related to the viscosity, surface tension, rheological properties, and cross-linking mechanisms of the hydrogels; it can be measured quantitatively using standardized tests. The primary method for assessing printability is to analyze the fiber diameter and pore size exhibited by 3D-printed hydrogel materials under varying printing pressures, printing speeds, and programmed fiber spacing.

Currently, the main method for enhancing the printability of hydrogels is to add easy-to-print components to the hydrogel to form a composite hydrogel (Chen et al., 2020). For example, nano-inorganic salt materials such as alginate are evenly incorporated into the hydrogel so that its overall microstructure remains stable and printable.

The first layer formation is a special consideration for hydrogel 3D bioprinting. It plays a crucial role in the formation and refinement of the overall 3D-printed structure. The first layer of printed bioink and the underlying structure maintain a larger contact angle, which is conducive to maintaining the upright and stable state of the bioink hydrogel material (Abdulghani and Morouco, 2019). The organic interaction between the 3D-printed hydrogel and the substrate facilitates both the overall positional anchoring of the 3D-printed structure and the effective avoidance of abnormal deformation and movement during the subsequent layer-by-layer printing process.

## 3 3D Bioprinting

The 3D bioprinting technology has become an extremely popular research direction in the field of medical tissue repair. This approach maximizes multiple biomaterials for tissue fabrication (Zamborsky et al., 2019). Bioprinting technology can also be described as the spatial patterning of living cells using bioinks. It involves three main components: a polymer solution, living cells, and a 3D printer. Bioprinting is known to be an additive manufacturing technique that assembles materials layer by layer and is, therefore, cost-effective. The popular 3D bioprinting techniques in orthopedics include extrusion, droplet/inkjet, and laser printing techniques, as well as other techniques such as printing based on stereoscopic laser scales such as UV (Huang J. et al., 2021). Other 3D scaffold manufacturing methods that are widely used in the field of tissue engineering include fused deposition modeling (FDM) and direct ink writing (DIW).

### 3.1 Extrusion-Based 3D Printing

**FIGURE 2:**
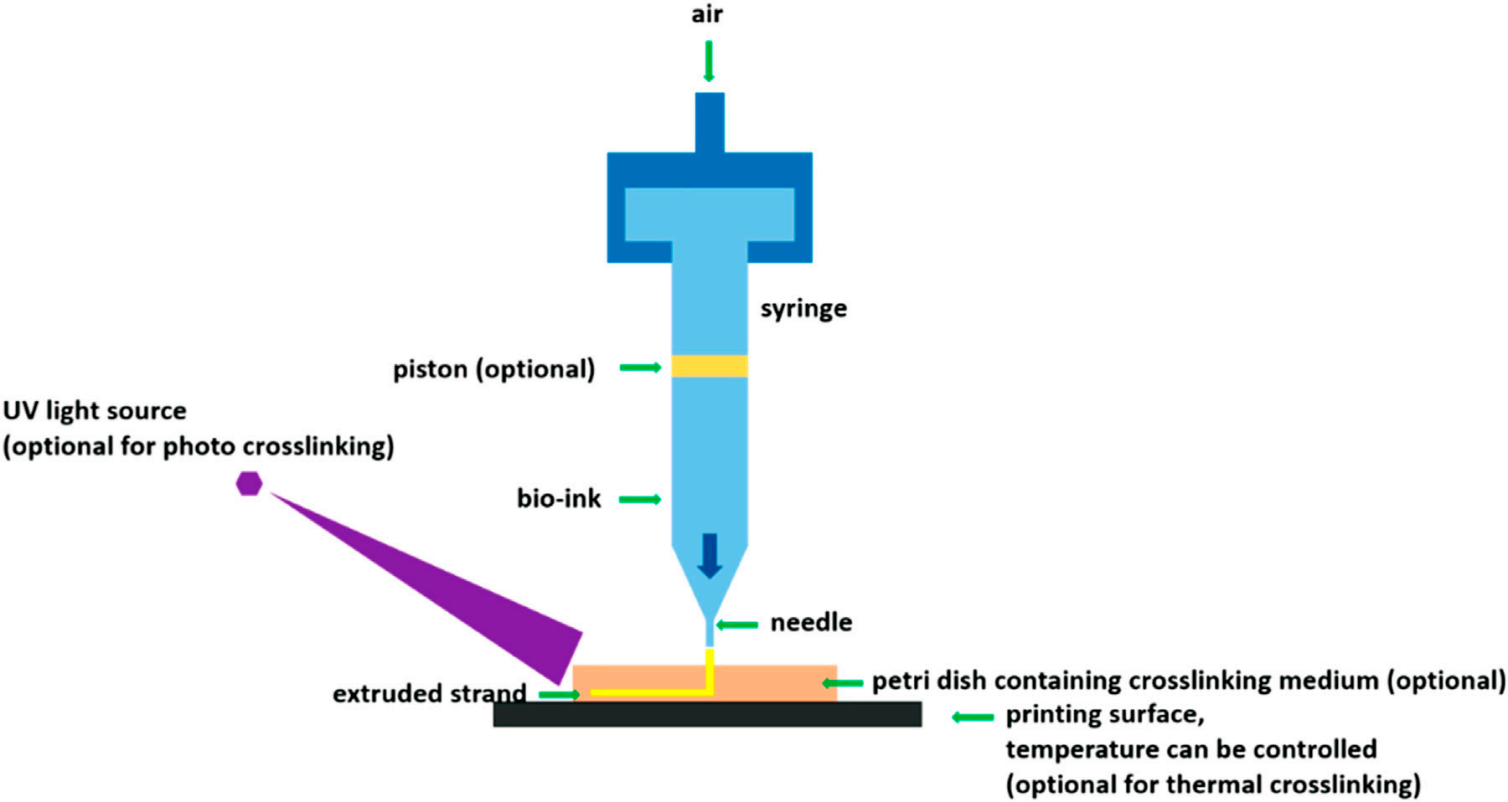
Extrusion-based 3D printing schematic. (Schematic of extrusion-based bioprinting using various crosslinking mechanisms (You et al., 2017). Reproduced from (You et al., 2017) with permission from Copyright 2021 MDPI).

Extrusion-based 3D printing is mainly based on the intrinsic pressure of the 3D printer; it uses nozzles to eject the hydrogel adhesive strips and arrange them on a 3D structure according to a computer-aided design model, relying mainly on the shear dilution of the bioink in this process (Dhawan et al., 2019). This technique can be driven in various ways, such as pneumatic, electromagnetic, or mechanical drives (Figure 2). This is achieved by using screws, pneumatic pumps, and pistons to distribute long filaments of hydrogel onto the substrate through nozzles, which become the skeletal structure of the bone or cartilage tissue. Subsequently, the stem cells encased in the hydrogel materials proliferate and differentiate in the 3D-printed structure, thereby generating an appropriate graft replacement.

Such techniques have high printing speeds and are also more conducive to the formation of porous structures such as hydrogels (Zhang et al., 2019). This 3D printing approach also offers unique advantages in printing high-cell-density bioinks and has the ability to build complex 3D structures with multiple cell types and materials. Nevertheless, the high shear force used during the printing process can easily damage and kill the cells (Zhang et al., 2019), which causes the elimination of more stem cells during the printing process and may impair viability of the surviving cells. Hence, the subsequent proliferation and differentiation processes may take more time or even fail to achieve the desired results (Cui et al., 2020). Furthermore, this 3D printing technology still has a low printing accuracy; therefore, the 3D graphics printed in this way may not precisely generate the desired bone and cartilage structures.

### 3.2 Droplet-Based 3D Printing

The droplet-based bioprinting method involves inkjet, acoustic droplet jetting, and microvalve bioprinting. It is mainly based on a traditional 2D bioprinter with a modified print head and print head control. This type of printing achieves a certain degree of control of the droplets produced by the printer, mainly by means of electric and ultrasonic energy, to eject droplets from the prepolymer solution onto the platform in a predefined pattern (Mora-Boza and Lopez-Donaire, 2018).

For example, thermal inkjet printers mainly use heat to generate pressure pulses in the print head in a short time, thus resulting in the ejection of bioink droplets (Turnbull et al., 2018). Other inkjet printing systems rely on piezoelectric crystals, which are mechanically stressed by the application of a voltage, which subsequently results in a change in shape. This approach further generates an acoustic wave, which creates sufficient pressure to eject the droplets from the nozzle.

This 3D printing technology has high printing accuracy and controllability because very small droplets can be manipulated as printing units. However, it also has apparent disadvantages in that the form of the bioink is severely restricted and must be in liquid form (Mora-Boza and Lopez-Donaire, 2018); additionally, it is difficult to control the droplet size consistently, there is uneven dispersion of cells in the droplet, and printer nozzles are prone to clogging.

### 3.3 Laser-Assisted 3D Printing

The laser printing approach, which is a relatively new technology, initiates the release of droplets mainly through the guidance of a laser. The main mode involves a two-layer approach, where the top layer is the energy-absorbing donor layer and the bottom layer is the bioink layer. When the top layer receives the laser pulse wave, bubbles are generated at the interface, thus pushing the droplets onto the substrate to achieve the release of the bioink. Using the high energy of the laser, the printer can operate without any direct contact with the model. This printing technology, similarly to the droplet-based printing technology, encompasses different modalities, including the Stereo Lithography Apparatus (SLA), Digital Light Processing (DLP), and two-photon polymerization (2 PP).

The SLA technology involves focusing a specific-wavelength and -intensity laser on the surface of a light-cured material, causing its sequential solidification from point to line and from line to surface, printing one layer, and then moving the lift table in the vertical direction by the height of that layer and subsequently curing another layer (Huang J. et al., 2021). The layers are thus stacked to form a three-dimensional structure. This technology displays high precision and speed and is already popular among laser-based 3D printing technologies.

The DLP modality is a variant of SLA. In this process, digital micromirror elements are used to project product cross-sectional shapes onto the surface of a liquid bright resin (Turnbull et al., 2018), light-curing it layer by layer. DLP 3D printing is faster than SLA owing to the slide/sheet-like curing of each layer. This technology is ideal for high-resolution molding.

Meanwhile, the 2 PP technology is a “nano-optics” application. This technology is similar to light-curing additive manufacturing, but 2 PP can achieve thinner layer thicknesses and *X* and *Y* axis resolutions between 100 and 200 nm (Mora-Boza and Lopez-Donaire, 2018). In contrast to the ordinary 3D printing optical polymerization with an ultraviolet laser, 2 PP uses a near-infrared long laser. As near-infrared wavelength photon energy, linear absorption, and Rayleigh scattering are low, and penetration in the medium is high, 2 PP enables the production of a wider variety of bone and cartilage replacement implants.

Laser-assisted 3D printing is highly effective for the utilization of bioinks with low viscosity. As a result, it only prints relatively thin structures. Although this printing method has high accuracy and resolution, its high cost and demanding requirements for bioinks limit its wide application.

## 4 Cartilage Repair and Reconstruction

3D printing based on hydrogels can solve many of the problems and shortcomings of the restorative treatment of articular cartilage injury that is currently used in mainstream clinical practice. First, bone marrow mesenchymal stem cells or cell clusters with differentiation capacity are added into hydrogels, such as chondrogenic cells (ACPCs), whose differentiation capacity and characteristics have been demonstrated by Riccardo et al. (Levato et al., 2017). These differentiated cell tissues are added into the biocarrier hydrogel, and then the hydrogel units that are wrapped with cell clusters are deposited into the already constructed cartilage design model. In this case, the initial skeletal shape and volume of the 3D-printed implant material are fixed at the beginning. These hydrogel materials basically mimic the extracellular matrix and microenvironment in the human body, wherein the stem cell clusters can naturally and rapidly divide and differentiate. Eventually, the cells constitute the volume and morphology of the scaffold by continuously differentiating and proliferating and eventually migrating to the various components of the scaffold (Vega et al., 2017), at which point the ideal hydrogel material will begin to degrade naturally. In order to better promote cell differentiation and proliferation, a series of cytokines, such as BMPs and TGF, can be added based on the liquid behavior of the hydrogel (Gibbs et al., 2016), in addition to suitable nutritional conditions, to further promote the differentiation and growth of cartilage tissue.

### 4.1 Single Material Hydrogels

Some natural hydrogel materials inherently have the ability to promote chondrogenesis, such as collagen; Wincent et al. showed that mesenchymal stem cells, articular chondrocytes, etc. can undergo specific chemical reactions with collagen, microcurrent change sensing, etc. (Maisani et al., 2018), thus promoting cellular differentiation and regeneration. In addition to alginate and collagen, there are a series of natural materials with high biocompatibility, such as agarose, fibrin (Noori et al., 2017), and hyaluronic acid (Noori et al., 2017).

Polysaccharide hydrogel materials (Sahai et al., 2021) play a crucial role in living systems. These abundant polysaccharides inherently possess ideal properties such as biocompatibility, biodegradability, and functional groups that contribute to simple chemical modifications for customizability, cytocompatibility, and organized macrostructural features, which makes them promising biomaterials (Radhakrishnan et al., 2017). Alginate is also a type of polysaccharide; specifically, alginate, or sodium alginate (in its original form) is an anionic polysaccharide of algal origin, consisting of mannoglycan aldehyde and gulonate units. The structure of alginate is similar to that of natural ECM, and properties such as good biocompatibility, high viscosity, and easy gelation make it an ideal material for 3D bioprinting (Radhakrishnan et al., 2017). Polysaccharide-based hydrogel materials are widely used in 3D hydrogel printing of cartilage and bone.

The biocompatibility of gelatin itself is due to the fact that the Arg-Gly-Asp (RGD) sequence in collagen is better preserved in gelatin (Gu et al., 2018), which allows gelatin to effectively promote cartilage tissue cell generation and migration.

Hyaluronic acid not only possesses excellent biocompatibility and the ability to promote chondrocyte differentiation and proliferation but also has good adhesion and ductility (Antich et al., 2020). Therefore, it is also used extensively in hydrogel-based 3D-printed cartilage repair techniques.

Apart from natural hydrogel materials, an increasing number of synthetic hydrogels are being used in large quantities for 3D printing of cartilage repair and reconstruction (Kundu et al., 2015). The utilization of synthetic hydrogel materials in cartilage repair and reconstruction has unique advantages (Lin et al., 2017; Yang et al., 2021).

### 4.2 Emerging Composite Hydrogels

To obtain more ideal bioprinting materials, researchers have combined different hydrogel materials to create composite hydrogel materials with more comprehensive characteristics and have made progress in 3D-printed cartilage repair engineering.

**FIGURE 3:**
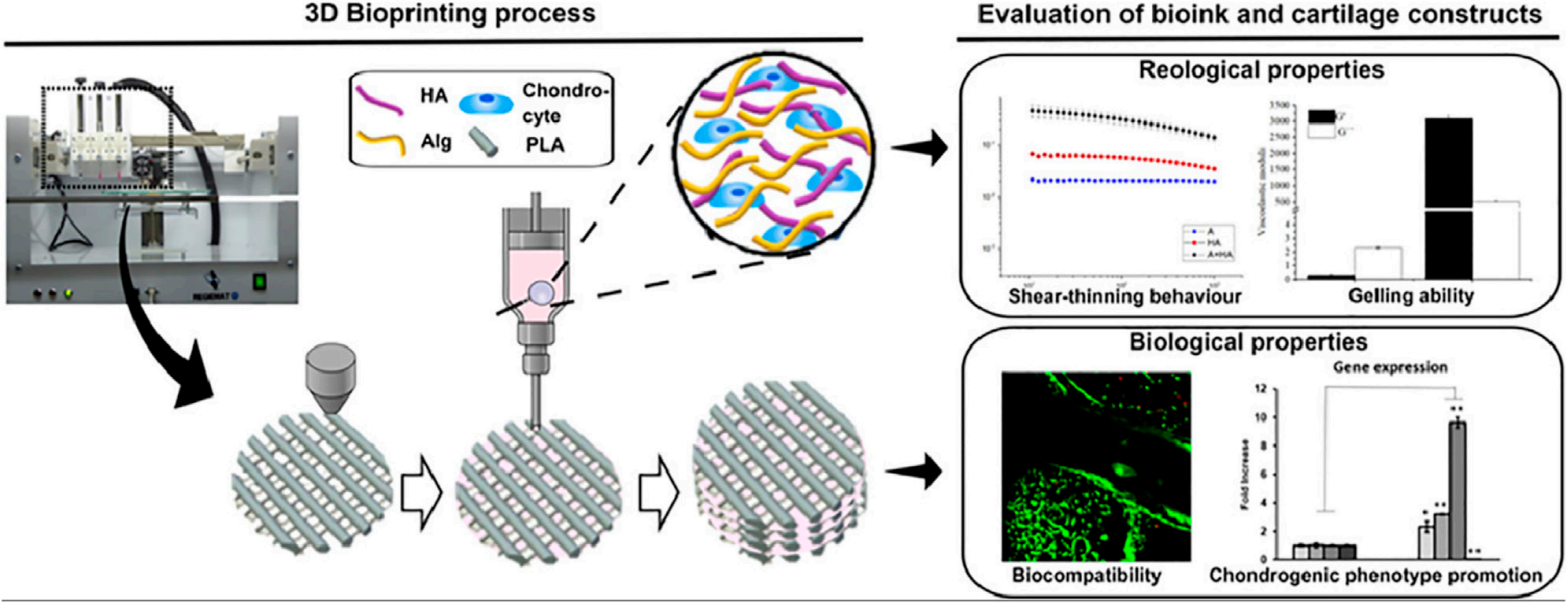
A new composite hydrogel material for the 3D printing of articular cartilage repair (Antich et al., 2020) (Reproduced from Creative Commons license).

Antich et al. combined hyaluronic acid with alginate to obtain a new composite hydrogel material for the 3D printing of articular cartilage repair (Antich et al., 2020). The authors analyzed the properties of these HA/PLA composite hydrogels, such as viscosity, degradability, cell viability; moreover, they performed the karyotype analysis of new cartilage formation, by combining hyaluronic acid with alginate in the presence of calcium ions to form physical cross-links (Figure 3).

Cartilage formation in hybrid 3D-bioprinted constructs after 1 month in culture. Quantitative analysis of type II collagen 1) and GAG 2) in the total extract per scaffold (in 1 ml). 3) Gene expression levels of hyaline-specific chondrogenic marker genes (COL2A1, ACAN, and SOX9), fibrotic maker (COL1A1) and hypertrophic marker (COL10A1) in bioprinted 3D hybrid construct (mean ± SD, *n* = 3, Student’s t-test, *p < 0.05, **p < 0.01). Reproduced from Creative Commons license.

Similar experiments for the development of bioinks were carried out by Mouser et al. They added methacrylated hyaluronic acid (HAMA) to a thermosensitive hydrogel composed of methacrylated poly [N-(2-hydroxypropyl)methacrylamide mono/diacrylate] (pHPMA-lac)/polyethylene glycol (PEG) triblock copolymer, to improve the printability of the bioink and guarantee the shape and volume of the generated cartilage tissue (Mouser et al., 2017). Moreover, they co-printed the bioink with polycaprolactone (PCL) to improve the mechanical stability of the bioink. Finally, the performance of the formulations was evaluated by *in vitro* incubation. The experimental results showed that the concentration of HAMA has a significant impact on the ability of chondrocytes to produce cartilage matrix, which shows a positive correlation at a certain concentration. However, an extremely high concentration can also lead to poor fibrocartilage formation. The final results of the experiment showed that the concentration of HAMA between 0.25 and 0.5% was the most favorable for the formation of the cartilage matrix. Moreover, the experimental results also showed that the synergistic printing of this triblock copolymer composed of thermosensitive hydrogel and PCL could produce 3D structures with mechanical strength almost identical to that of natural cartilage.

These studies mainly focused on improving the biocompatibility of 3D-printed hydrogels. Studies have also considered improving the physical properties of 3D-printed hydrogels to make them more suitable as printed bioinks. For example, Olate-Moya et al. intended to improve the mechanical properties and processability of hydrogels by compounding them. For this purpose, they combined the nanoparticle material graphene oxide with alginate (Olate-Moya et al., 2020) and added chondroitin sulfate from glycosaminoglycans to ensure the histocompatibility of the hydrogel material. The study and analysis of the characterization of the newly synthesized bioink, the water content and swelling rate of the 3D-printed scaffold, the rheological properties of the ink, the mechanical properties of the hydrogel, the biological interaction between the scaffold and BMSCs, and the histological and cytological studies of the new cartilage revealed several benefits. They showed that the new hydrogel material has enhanced printability, superior rheological properties, and a robust scaffold morphology retention. In addition, the new material also has good histocompatibility owing to the addition of chondroitin sulfate.

Most of the current trials and studies are based on this model. When natural or artificial hydrogel materials with different properties are combined through different cross-linking methods, a composite hydrogel material with all the superior properties of its components is obtained. Therefore, this model has the potential to create hydrogel materials with good biocompatibility, good mechanical properties and printability, and the ability to promote the proliferation and differentiation of cartilage.

You et al. targeted calcified cartilage in the deeper layers of articular cartilage, which is an important transition location for the repair of calcified cartilage; repair of this damage is difficult to achieve with most current regenerative engineering as the osteochondral interface is critical for maintaining the structural integrity of the joint (Yang et al., 2017). Therefore, in order to fully mimic the structure and generation process of calcified cartilage, homogeneous hydroxyapatite was mixed with alginate hydrogel, the resulting hydrogel was used for 3D bioprinting, and the printed material implanted subcutaneously in mice (You et al., 2018). The cellular activity, proliferation, and secretion of cartilage matrix were evaluated in the experiment, and the potential for cartilage tissue mineralization was quantified by assaying alkaline phosphatase activity. The rheology and print fidelity of the composite formed hydrogels were also considered. The final experimental results showed that the addition of HAP to the alginate hydrogel did not impair the activity and proliferation capacity of the cells, and it did not affect their secretion of cartilage matrix. Furthermore, it stimulated the secretion of mineralized matrix by chondrocytes, thus promoting mineral deposition *in vivo*, provided that the HAP particles were well dispersed. This experimental result illustrates the promising application and characteristics of this material in promoting calcified cartilage formation and ensuring the integrity of bone and joint repair.

Once the articular cartilage generated by hydrogel-based 3D bioprinting has been printed, its clinical application is another significant challenge. Only a few are currently used for humans. Preliminary tests have been performed in small animals, and very few preliminary applications have been performed *in vivo* in large organisms. In a more recent study by Shim et al., 3D-printed chondrocytes were implanted into the knee joints of rabbits, where evidence of fusion of bone and cartilage growth, the generation of new joints, and the production of cartilage surface membranes was observed (Ge et al., 2009). Zhang et al. conducted an *in vivo* study in rhesus monkeys, where electron beam melting was used to develop a porous titanium cage filled with simvastatin/poloxam 407 hydrogel and bone inward growth and spinal fusion were evaluated. This experiment was performed in larger animals (monkeys), providing a preliminary lead for experiments in humans (Zhang et al., 2020). These experiments and studies illustrate the massive potential of hydrogel-based 3D-printed cartilage structures implanted *in vivo* as a replacement therapy for bone joints, thus offering a new approach to articular cartilage repair.

## 5 Bone Repair and Reconstruction

### 5.1 Special Nature of Bone Requirements

Infections caused by bone and joint injuries not only damage the articular cartilage but also inevitably affect the bone when the lesions have progressed to a certain level or when they are in a very specific location. Osteoarthritis, fractures, traumatic bone tumors, and other conditions directly affect the quality of long bones. Therefore, 3D-printed hydrogels have been extensively investigated, and to some extent, used for the repair and reconstruction of bone tissue (Feng et al., 2021).

The bone has unique characteristics compared to cartilage, and therefore also has some unique requirements for 3D-printed hydrogel materials, including some functions and characteristics of higher standards (Hernandez et al., 2017). First, 3D-printed hydrogels for bone tissue repair and reconstruction need to possess higher strength and hardness compared to that used for cartilage (Chen C. Y. et al., 2021). 3D-printed hydrogels have sufficient mechanical strength not only for maintaining human mechanics but also for maintaining the mechanical stability of the 3D-printed structure itself (Huang K.-H. et al., 2021). The angiogenesis of bone tissue is more important than the cartilage tissue (Son et al., 2015). The structure of bone tissue is complex, and it is mainly composed of various skeletal cells and blood vessels that penetrate them. The blood vessels provide the necessary oxygen and nutrition for bone tissue cells. Therefore, it is essential for 3D-printed hydrogels to possess pro-angiogenic properties in bone tissue.

### 5.2 Composite 3D-Printed Hydrogels

By organically combining different hydrogel materials, hydrogel materials can possess good mechanical strength while combining the original biocompatibility and viscosity properties. The current mainstream approach to increase the mechanical strength of hydrogels is to add inorganic molecules, such as silicates, to the hydrogels. Liu et al. developed a composite hydrogel material based on gelatin and alginate and further added nanoscale silicate materials to the original composite hydrogel material (Liu et al., 2020), with the aim of retaining the original properties of the composite hydrogel and further increasing the mechanical strength. Hydration tests, microstructural and elemental analyses, rheological analyses, and uniaxial compression tests were performed to evaluate the newly synthesized hydrogel materials, and cryo-transmission electron microscopy and Fourier transform infrared spectroscopy (FT-IR) were used specifically for mechanical strength and cell proliferation assays and *in vitro* osteogenesis analyses. An *in vivo* model of cranial defects in rats was also established to evaluate the *in vivo* experimental effects of this hydrogel material. The final results showed that the mechanical strength of the hydrogel material was significantly enhanced by the addition of nanosilicate to the composite hydrogel material, and the excellent properties of the hydrogel were retained.

Besides silicates, research has been conducted on carbon materials (Cheng et al., 2018); graphene oxide materials are of great use in bone and cartilage repair (Cheng et al., 2020). For example, Dr. Qi Zhao’s team prepared a new composite hydrogel material by preparing serine as a methacrylate hydrogel material and then combining it with graphene oxide. This hydrogel material was used for the repair of rat cranial bone in certain *in vivo* experiments. During the experiments, the density of the generated bone was assessed by X-ray, histological, and cytological analyses, and finally, RNA sequencing analysis was performed for a deeper assessment (Qi et al., 2020). The experimental results showed that the addition of graphene oxide to the hydrogel material significantly increased the mechanical strength of the hydrogel structure; moreover, the addition had no impact on the biocompatibility of the hydrogel. However, the addition of graphene oxide reduced the degradability of the hydrogel material to some extent. In a comparative analysis with the control group, the effectiveness of the hydrogel for osteogenic treatment was demonstrated by measuring bone density and analyzing bone tissue histology. A further RNA sequencing was performed to analyze the activation of the relevant osteogenic cytokine signaling pathways in the context of the hydrogel treatment. In the experimental results, it was observed that the relevant signaling pathways such as BMP showed more activities, thus reinforcing the potential of the new composite 3D-printed hydrogel as a bone tissue repair material. There are many other compounds that can be used to enhance the mechanical strength of hydrogels, and many similar hydrogel materials have been explored and fabricated based on these compounds (Pathmanapan et al., 2020).

In addition to the pressure resistance, the mechanical strength of hydrogel materials also includes tensile resistance. It is important that the hydrogel structure maintains its original physical structure and morphology in the face of external pressure and possess good tensile resistance against stretching (Bouler et al., 2017). Therefore, double network gels (DN gels), which are composite hydrogels formed by combining two polymers with different physical properties, have been studied and developed. Here, one is a rigid network and the other is a flexible network; such a structure ensures that the hydrogel material maintains its structural resistance to pressure and also ensures its resistance to stretching. Bi et al. combined two materials, chitosan and polyvinyl alcohol (PVA), to form a double network hydrogel material; to further enhance its mechanical strength and osteogenic differentiation, hydrogel surface mineralization was introduced, and based on this, the hydrogel material was combined with inorganic compounds. This was done by repeatedly soaking the double-mesh hydrogel in aqueous solutions of calcium chloride and potassium hydrogen phosphate alternately, which yielded mineralized crystals on the surface of the hydrogel (Bi et al., 2019). The surface mineralized double-network hydrogel material obtained through this process was evaluated under several physical conditions, along with its histological and cytological analyses. These analyses demonstrated that this material clearly possesses a certain degree of enhanced physical and mechanical strength and better histocompatibility and osteogenic differentiation induction.

The rheological properties of hydrogels have also been a significant challenge in the field of 3D-printed bone tissue engineering. An ideal hydrogel material ensures that the encapsulated cells can be uniformly distributed and should migrate efficiently for growth during the 3D printing process. To address this challenge, Bendtsen et al. explored novel hydrogel composites by experimenting with two natural and synthetic materials, alginate and polyvinyl alcohol, to form a base composite hydrogel, and then adding hydroxyapatite to the hydrogel material as a base (Bendtsen et al., 2017). Hydroxyapatite (HA) is the main inorganic component of natural bone (Yao et al., 2021), and its incorporation into polyvinyl alcohol (PVA) suspensions was used to increase the viscosity of the hydrogel formulation and increase the biocompatibility and osteoconductivity of the printed scaffold. A variety of solution formulations was also used for the study to identify an optimal formulation system. The final results confirmed that the composite hydrogel material incorporating calcium hydroxyphosphate has good viscoelasticity at the appropriate concentration and is fully adaptable to the extrusion process in 3D printing and can later form a high-fidelity scaffold structure. The hydrogel provides favorable conditions for the regeneration of bone structures and has the ability to promote bone tissue generation *in vitro*.

Therefore, it can be seen that combining organic hydrogel materials with good biocompatibility and inorganic compounds with certain mechanical strength in an appropriate way (Heid and Boccaccini, 2020) can effectively combine the advantages of both materials and create hydrogel materials with more promising applications in the field of bone tissue repair and reconstruction. These inorganic compounds are mainly the aforementioned silicate-based inorganic materials, calcium phosphate-based inorganic materials (Dhivya et al., 2015), and newly discovered inorganic materials containing metallic strontium. The inorganic materials containing metallic strontium can integrate with bone hydroxyapatite and reduce bone resorption by increasing the number of bone formation sites and decreasing the number of active osteoclasts. Therefore, strontium-doped bioactive glasses, hydroxyapatite, and calcium phosphate (scaffolds) show great promise as bone graft replacement materials.

The microscopic hydrogel materials (Chen et al., 2020) have also been used extensively in hydrogel-based 3D-printed bone repair and reconstruction experiments. The charged properties of the hydrogel microstructure, volume size, and shape of the microstructure are important determinants of bone tissue generation. Prof. Cui Zhang’s group focused on studying composite hydrogels with nanomaterials, starting with chitosan as the most primitive raw material, and used methylated polyethylene glycol chitosan as the base plate material, wherein they introduced montmorillonite (MMT). The MMT particles are usually plate-shaped with a thickness of approximately 1 nm and a diameter of approximately 0.2–2 μm; the repeating structural unit of MMT consists of an alumina octahedral plate sandwiched between two silica tetrahedral layers (Cui et al., 2019). Because the surface of MMT is dominated by oxide anions, its overall surface charge is shallowly negative, which facilitates the mixing of this nanohydrogel material with cationic agents. Therefore, the introduction of this material into the hydrogel enables the nanostructure of the composite hydrogel to be cross-linked and sparsely porous. This porous structure can mimic the microenvironment of bone proliferation and differentiation in a highly reductive manner; this ensures that the composite 3D-printed hydrogel nanomaterial is highly induced by osteogenic differentiation. Therefore, by focusing on the development of 3D-printed hydrogel microstructures, the improved hydrogel materials from the molecular and cellular levels of osteogenic differentiation could be used in a large number of bone tissue engineering applications in the future.

Another major challenge in the field of bone tissue repair is the repair of large segmental bone defects that exceed a critical size (Chen et al., 2019). Yu et al. have made some attempts in this field by applying 3D printing hydrogel technology. The primary materials that were used included preosteoblast-derived matrix (Yu et al., 2020) (a special decellularized extracellular matrix material) and gelatin methacrylate (GelMA) hydrogel. Bone marrow mesenchymal stem cells (BMSCs) were inoculated to grow on the prepared composite hydrogel material to study properties for *in vitro* experiments. *in vivo* experiments were also performed on rabbits by artificially creating large-stage bone defects of 15 mm and using the implants generated from this hydrogel material for *in vivo* experiments. Their experimental results showed that *in vitro*, pODM exhibited some degree of chemotaxis and osteogenic induction of bone marrow mesenchymal stem cells (BMSCs). The implantation of pODM/gelatin methacrylate (GelMA) structures as engineered periosteal bone substitutes effectively repaired critical-sized segmental bone defects in the radius of rabbits. What’s more, the hydrogel material of the extracellular matrix we have already mentioned in the previous introduction of hydrogel material itself is very loose and porous, which facilitates cell growth and migration.

### 5.3 Angiogenesis

An additional feature required for hydrogel materials for bone tissue reconstruction and repair is the ability of angiogenesis, as blood supply is the primary factor for the survival of new bone tissue without necrosis (Kuss et al., 2018). Current research has proposed two main strategies for generating vascular tissue; one is the artificial creation of vascular tissue through the distribution of vascular endothelial cells and the other is the formation of neovascularization through the self-assembly of the organism itself (Koç et al., 2014; Deng et al., 2017; Lin et al., 2019). Professor Batzaya Byambaa’s group has studied and explored several levels and dimensions of angiogenesis in bone tissue engineering. First, to study the structure of the microenvironment, they created cylindrical bio-skeletal units of hypomethacrylic gelatin hydrogels and combined the hydrogel material with vascular endothelial generating factor (VEGF) to form gels of conjugated compounds (Byambaa et al., 2017). This hydrogel was then applied to bone tissue engineering, and its osteogenic induction and angiogenic ability were evaluated by cytological and histological methods. The results showed that after microenvironmental modifications and the addition of VEGF, this particular hydrogel had a good angiogenic effect, which improved bone tissue recovery and reconstruction, resulting in a more complete and comprehensive bone tissue repair. In addition, the hydrogel material with photothermal effect, which was mentioned before, has good potential in angiogenesis by quantitatively regulating the temperature of the hydrogel and thus controlling the physical state, microenvironmental temperature, and release of embedded cytokines.

## 6 Anti-infection Properties of 3D-Printed Hydrogels

Currently, 3D-printed hydrogels are one of the most popular materials for bone tissue engineering, and their antibacterial and anti-infection effects are excellent properties. Furthermore, studies on the antimicrobial properties of hydrogels are also one of the most popular research directions today. A significant portion of articular cartilage and bone damage is caused by infection and inflammation, and severe infection can cause irreversible damage to the bone and joint. Other causes of osteoarthritic injury, such as external mechanical violence, inevitably cause secondary infections. There are also a large number of medical operations, cartilage and bone repair and reconstruction, and the implants themselves may cause infection, and thus, the problem of infection will continue throughout the process of bone and joint repair and reconstruction. The hydrogel materials currently studied in the laboratory have shown good potential in terms of antibacterial and anti-infective properties (Li et al., 2017).

The hydrogel materials with antimicrobial activity are divided into three main categories. The first category is hydrogel materials containing inorganic antimicrobial metal ions or metal oxide nanoparticles, such as silver (Lu et al., 2016), copper and other metal ions, zinc oxide, nickel oxide, and other metal oxides. The second category is hydrogel materials containing antibiotic antimicrobial agent drugs, which are mainly combined with hydrogel and wrapped inside the hydrogel material, relying on the liquid behavior of the hydrogel, which is released at the appropriate time to play an antibacterial and anti-infective role (Chen Z.-Y. et al., 2021). The last category is the hydrogel whose material itself has antibacterial and anti-infective ability, which is rare. It also has very obvious advantages; it is bactericidal and effective with almost no toxic side effects.

### 6.1 Addition of Antimicrobial Inorganic Metals

The antibacterial ability of silver-containing materials has been widely demonstrated in clinical practice, and silver-containing dressings are now commonly used in clinical surgery to treat wounds and prevent infection. Thus, biohydrogel materials containing silver ions in nanoparticles (Xu et al., 2018) are a common combination of anti-infective metal ions and hydrogel materials, and nanoparticles of silver ions can bind to natural hydrogel materials such as polysaccharides. Furthermore, they possess the ability to bind to many synthetic hydrogel materials. Silver ions have good efficacy in dealing with the anti-infective effects of Gram-negative bacteria. In a recent study (Ribeiro et al., 2017), Marta Ribeiro et al. incorporated particles of silver and gold nanoparticles into filamentous protein/hydroxyapatite nanogels to synthesize 3D-printed hydrogel materials with good antibacterial properties and the ability to promote bone tissue repair. During their experiments, they characterized the presence of AgNPs and AuNPs in the hydrogels using UV spectrophotometry, transmission electron microscopy, and thermogravimetric analysis. The *in vitro* antibacterial studies showed that hydrogels containing AgNPs and AuNPs exhibited significant inhibition of both Gram-positive and Gram-negative bacteria. The cytocompatibility studies using osteoblasts showed that the hydrogels had a significant effective antibacterial ability at a concentration of 0.5% for AgNPs, but for AuNPs and the hydrogels containing AuNPs were effective as antibacterial materials at almost all concentrations within the normal range without affecting cell behavior. In this experiment, it can be seen that besides silver ions, gold ions also have good antimicrobial properties. Some other experiments demonstrated that copper ions can also be added to 3D-printed hydrogel materials for antimicrobial activity (Zou et al., 2020).

In addition to these metal ions, researchers have also explored emerging rare metal ion materials, such as strontium metal (Tommasi et al., 2016) and lanthanum metal, which may possess a greater antibacterial potential and may also have more osteogenic activity. Moreover, when these metal ions are compounded with other hydrogel materials, the newly generated hydrogel materials may have a better ability to promote bone and cartilage tissue production in addition to antibacterial properties. For example, Professor Chen et al. introduced lanthanum metal ions into double-physical cross-linked hydrogel materials. In the experiments, the experimentalists created a network of dual physically cross-linked hydrogel materials from two materials, alginate, a natural hydrogel material, and polyvinyl alcohol, a synthetic material, and then added lanthanum metal (Hu et al., 2017). The mechanical properties, luminescence properties, biocompatibility, and antibacterial activity of this composite hydrogel were comprehensively evaluated in the experiments. It was found that this hydrogel material has excellent compressive and tensile resistance and excellent biocompatibility due to the double physical cross-linked structure, and most importantly owing to the introduction of lanthanum metal ions. These ions not only possess good photosensitive properties but also facilitate the detection and regulation of the hydrogel material in various biological microenvironmental situations; they also have a good antibacterial effect (Yang et al., 2016). Compared to silver ion materials, lanthanum metal ions showed good inhibitory effects on *Staphylococcus aureus* and *Escherichia coli* in the experiments. The discovery of this unexpected antibacterial effect may be of great reference value for the development of antibacterial hydrogels containing metal ion nanoparticles.

In addition, inorganic materials and metal oxide particles possess good bactericidal effects, mainly through the mechanism of photocatalytic reaction to kill microorganisms in a relatively short period of time, and zinc oxide is currently the most widely used metal oxide. It has shown good bactericidal activity in experiments and has almost no cytotoxicity. The antimicrobial properties of the material were investigated by experimentalists who formed ZnO nanorods *in situ* in a cross-linked carboxymethyl chitosan (CMCh) matrix, treated the CMCh hydrogels with zinc nitrate solution, and then oxidized the zinc ions with an alkaline solution to successfully prepare antimicrobial carboxymethyl chitosan/ZnO nanocomposite hydrogels (Wahid et al., 2016). We focused on the point where the researchers investigated the antibacterial activity of CMCh/ZnO hydrogels against *Escherichia coli* and *Staphylococcus aureus* by CFU assay. The experimental results showed that the synthesized CMCh/ZnO nanocomposite hydrogels exhibited excellent antibacterial activity.

### 6.2 Addition of Antibiotics

The incorporation of antibiotics into hydrogel materials is also a common method used. Currently, the primary methods include the use of hydrogel material as a carrier for antibiotic drugs. Here, antibiotics are added to the microstructural units of the hydrogel to be released at the right time, and also by surface nanomaterial engineering, wherein the antibiotic drugs are infiltrated on the surface of the hydrogel. Some of the antibiotics that are often incorporated into hydrogel materials in research include ciprofloxacin and vancomycin (Jung et al., 2019). Salma et al. constructed a nano-hydrogel material containing doxycycline with the aim of enhancing its anti-infective ability by using 3D-printed hydrogels for drug delivery. The base material of this hydrogel material is a composite of three materials: gelatin, polyvinyl alcohol, and hyaluronic acid. The specific method used was to first prepare doxycycline/hydroxyapatite nanoparticles (DX/HAp) by mixing followed by solution centrifugation, etc., and then by mixing the manufactured particles into polycaprolactone (El-Habashy et al., 2021), and finally, fusing them with gelatin and hyaluronic acid materials to form doxycycline 3D-printed composite hydrogel material with relatively uniform dispersion. The *in vitro* experiments performed on this hydrogel material were aimed at determining the physicochemical characterization and *in vitro* drug release ability of the hydrogel material. The *in vivo* experiments were conducted to construct a rabbit injury model and to repair and reconstruct bone tissue using this 3D-printed hydrogel material, which was eventually analyzed by histological and cytological measurements of the repaired tissue. The experimental results also largely met the expectations because this newly synthesized hydrogel material not only has good rheological properties and biocompatibility, but most importantly, it can release doxycycline both *in vivo* and *in vitro*.

In addition to inorganic metal ion oxidation and antimicrobial agents, scientists have also explored other substances added to hydrogel materials to play a certain antimicrobial role. For example, some biological extracts with special antibacterial effects, such as curcumin, honey, and lignin (Yang et al., 2018), as well as carbon materials and lysozyme (Wu et al., 2018), have shown some degree of antibacterial and antimicrobial effects against both *S. aureus* and *E. coli* when added to 3D-printed hydrogels.

### 6.3 Inherent Antibacterial Activity

The main hydrogel materials with inherent antimicrobial activity are synthetic polymeric antimicrobial materials (Chang et al., 2021), for example, redox-generated iron-based silane macromolecular hydrogels and some materials containing antimicrobial peptides, which are still relatively rare and have not been widely studied or applied in the field of orthopedics.

## 7 Current Shortcomings of 3D-Printed Hydrogels

Hydrogel-based 3D printing technology has been extensively explored, and impressive results have been achieved in experimental research. However, there are still several shortcomings in this technology that need to be addressed.

### 7.1 Insufficient Mechanical Strength

In addition to the diversity of hydrogel properties and the mechanical strength of hydrogels alone, there are a few other problems that need to be overcome. The mechanical strength of hydrogels includes resistance to compression, tensile properties, and shear dilution capabilities. Firstly, the 3D-printed hydrogel bio-skeleton should possess the ability to retain its own morphology and structure and resist pressure from outside. Moreover, the hydrogel could also be stretched and pulled during the process of 3D printing, and the ductility of the material with compressive ability may not be as ideal as the compressive ability. A hydrogel material with sufficient and comprehensive capabilities to cope with various experimental and clinical environments is needed for practical clinical applications.

### 7.2 Angiogenic Capacity

The 3D-printed bone tissue engineering using hydrogels needs to be further investigated and solved by establishing vascular networks in the generated bone and cartilage replacement tissues. The survival and long-term maintenance of new tissues can only be ensured by establishing a vascular network in new tissues and structures to maintain the blood supply to the new tissues. Currently, there are relatively poor coping strategies to solve the problem of vascular network generation and establishment, and it is still in the preliminary experimental and testing stage (Chen et al., 2018). Therefore, a certain volume and scale of 3D-printed bone tissue replacement have hardly been tested because this problem of vascular generation and vascular network establishment needs to be solved before we can further consider a certain scale of regenerated tissues and structures.

### 7.3 Insufficient Intrinsic Antibacterial Activity

The sterility and antibacterial ability mentioned previously are also worthy of attention. Several existing materials with antimicrobial activity are not satisfactory in other aspects of performance and have not received widespread attention. Therefore, the main approach taken to solve the antimicrobial problem is the addition of metallic substances or antibiotics, which are antimicrobial substances in hydrogel materials; because they are added substances, they have to consider the problem of uniform dispersion and long-term release similar to the cytokines mentioned previously. In addition to the antimicrobial properties, we must also ensure the sterility of the 3D-printed hydrogel material itself during the whole process and not only in the experimental environment. For practical applications, the process flow of the production and the surgical self-contained whole process to ensure the sterility of the 3D-printed implant material needs to be chosen according to the hydrogel’s own characteristics.

### 7.4 Degradability and Cytotoxicity of Degradation Products

The degradability and cytotoxicity of hydrogels are major criteria for consideration of hydrogels, and are one of the current shortcomings. Once the hydrogel material constitutes the 3D-bioprinted biological skeleton, the real need for clinical treatment is to implant the new bone and cartilage tissues formed into the human body to achieve clinically effective bone and cartilage tissue repair and reconstruction (Ma et al., 2018). However, whether and when the hydrogel material will eventually need to be degraded in this process (Gao et al., 2019), and whether the material generated after degradation is cytotoxic are questions that must be considered. If we create a repair material that is implanted in the human body for a long time together with the hydrogel, then we need to consider the maintenance of the hydrogel in the body. When the cartilage and bone cells have proliferated and migrated sufficiently in the bio-scaffold material to constitute the whole tissue, the hydrogel is no longer needed to maintain the structure. Further concerns include the degradation of hydrogels and how we should regulate the time of its degradation in order to avoid degradation before tissue repair is generated (Gao et al., 2019) or a delayed degradation occurs. It is also important to note that even though most hydrogels are biocompatible and almost non-cytotoxic, once decomposed, whether the decomposition products can be guaranteed to be absolutely non-cytotoxic and persistent in the human body are issues that need to be further explored and considered.

### 7.5 Lack of Adequate *in Vivo* Experiments

There have not been sufficient *in vivo* experiments, and most existing ones do not involve 3D-printed implants. Most of these *in vivo* experiments have been performed on small animals such as mice and rats, with some degree of implantation on the surface of the body, and there have been no real cases of repair and replacement of a section of bone or a specific joint. One of the main *in vivo* experimental models used is the repair of cranial defects in mice; however, there is a difference between the repair and reconstruction of cranial bones and the repair of knee and hip injuries. In addition to the lack of further experiments on large animals, in addition to mice, we can also see the establishment of *in vivo* models of rabbits (Jiang et al., 2021), lack of *in vivo* experiments, and models of large mammals such as pigs and cattle, and thus, the practical application of 3D-printed hydrogel *in vivo* still should be comprehensively studied.

### 7.6 Lack of Systematic Scientific Consideration and Evaluation Criteria

The use of 3D-printed hydrogel materials in clinical applications also requires technical, regulatory, and ethical considerations and justifications. First, a set of strict manufacturing and use standards must be established for 3D-printed hydrogel materials to ensure the reliability and safety of the materials and implants themselves. The safety of the implant during implantation and the certainty of its repair ability need to be ensured. The use of this technology in the clinical setting will certainly be challenged by ethical considerations (Abbadessa et al., 2016b). In addition to a rigorous ethical review, we need to strictly regulate and review the scope of use of hydrogel 3D printing as a technology.

## 8 Outlook and Conclusion

The combination of bioprinting and (Kang et al., 2015) computer-aided algorithms, which allows the computer to recognize and analyze the CT, MRI, and other examination results by inputting them into the computer, and eventually generate a personalized design that fits the patient’s actual situation through built-in algorithms, is a key focus area in hydrogel-based 3D printing technology. Such a combination can effectively screen and select a suitable hydrogel material for the case and use it for appropriate reconstruction. The computer recognizes and analyzes the results of CT and MRI examinations, and eventually generates a personalized design that is tailored to the patient’s situation through built-in algorithms.

The 3D bioprinting with electronic computers combined with various control systems to achieve high precision is a popular future direction for the use of 3D-printed hydrogel materials at the micron or even nanometer level (Xu et al., 2018), thereby allowing a more favorable formation microenvironment for bone tissue.

In the future, it is almost certain that 3D bioprinting will transition into 4D bioprinting, which would involve the addition of a new dimension to the original one, that is, time. The shape and function of hydrogels can follow time, mainly referring to the hydrogel materials that are sensitive to the response to external stimuli. Control of hydrogel materials by changing external conditions such as light intensity, temperature (Ji et al., 2020), pH value, magnetic field, and electric field (Mei et al., 2021) has been achieved. Based on further in-depth research on this aspect, future hydrogel materials could possess memory functions (Abbadessa et al., 2016a); they could be able to adjust their physicochemical properties on their own, following the external environment and our objective needs.

However, there are many shortcomings that need to be addressed, such as more comprehensive and stable mechanical strength. Single hydrogel materials have distinctive qualities, but they are not sufficiently comprehensive; for example, gelatin, hyaluronic acid, and other materials with high biocompatibility and osteogenic–chondrogenic induction (Daly et al., 2016) have low mechanical strength (Ahlfeld et al., 2017). Furthermore, materials such as alginate and polyvinyl alcohol lack the ability to induce cell proliferation and differentiation as much as gelatin and hyaluronic acid; hence, certain cytokines and other substances need to be added to simulate the extracellular microenvironment to the possible extent. Furthermore, hydrogel materials with good intrinsic antibacterial ability are still very rare, and the antibacterial properties of the few existing ones are yet to be fully evaluated. Finally, the composite hydrogel preparation process is sophisticated and expensive. Although there is a wide variety of composite hydrogels, none of them have been widely accepted because they do not meet the standards specified for clinical use.

Therefore, we can see that 3D-printed hydrogel materials in orthopedics, especially in the field of bone and joint repair, possess a very wide range of potential applications and prospects, and a considerable part of the results have been achieved in experiments. Some researchers have also explored the clinical applications. Many materials have the ability to induce cellular osteogenesis into cartilage differentiation and proliferation; after a certain degree of improvement, they possess a certain degree of mechanical strength and antibacterial activity. Thus, it is bound to be a popular focus of future research. It is believed that after more research and future discoveries, 3D-printed hydrogel materials will be applied in the near future in osteochondral tissue repair and reconstruction to solve bone and joint injury problems.
